# Enhancing the Machining Performance of Nomex Honeycomb Composites Using Rotary Ultrasonic Machining: A Finite Element Analysis Approach

**DOI:** 10.3390/ma17092044

**Published:** 2024-04-26

**Authors:** Tarik Zarrouk, Jamal-Eddine Salhi, Mohammed Nouari, Abdelilah Bouali

**Affiliations:** 1 CREHEIO (Centre de Recherche de L’Ecole des Hautes Etudes d’Ingénierie), Oujda 60000, Morocco; 2 CNRS, LEM3, IMT, GIP InSIC, Université de Lorraine, F-88100 Saint Dié Des Vosges, France; mohammed.nouari@univ-lorraine.fr; 3 Departement of Mathematics, Saveetha School of Engineering, SIMATS, Chennai 602105, India; j.salhi@ump.ac.ma; 4 Laboratory of Energetics (LE), Faculty of Sciences, Abdelmalek Essaadi University, Tetouan 93000, Morocco

**Keywords:** modeling, Nomex honeycomb composites (NHCs), rotary ultrasonic machining (RUM), surface quality, chip size, stress distribution

## Abstract

Nomex honeycomb composites (NHCs) are commonly used in various industrial sectors such as aerospace and automotive sectors due to their excellent material properties. However, when machining this type of structure, problems can arise due to significant cutting forces and unwanted cell vibrations. In order to remedy these shortcomings, this study proposes to integrate RUM (rotary ultrasonic machining) technology, which consists of applying ultrasonic vibrations along the axis of rotation of the cutter. To fully understand the milling process by ultrasonic vibrations of the NHC structure, a 3D numerical finite element model is developed using Abaqus/Explicit software. The results of the comparative analysis between the components of the simulated cutting forces and those from the experiment indicate a close agreement between the developed model and the experimental results. Based on the developed numerical model, this study comprehensively analyzes the influence of the ultrasonic vibration amplitude on various aspects, such as stress distribution in the cutting zone, chip size, the quality of the machined surface and the components of the cutting force. Ultimately, the results demonstrate that the application of ultrasonic vibrations leads to a reduction of up to 50% in the components of the cutting force, as well as an improvement in the quality of the machined surface and a reduction in the size of chips.

## 1. Introduction

The aerospace and aeronautics industries are strongly focused on the search for solutions offering both lightness and exceptional performance, particularly in extreme environments and conditions [1]. Nomex honeycomb composites (NHCs) meet these standards by providing high specific strength and stiffness [2,3,4]. This helps reduce weight while maintaining adequate strength and rigidity. Regarding their physicochemical properties, NHCs exhibit satisfactory characteristics in terms of corrosion resistance, fire resistance and dielectric properties, thus improving their performance under extreme requirements [5,6]. Machining the Nomex honeycomb core represents a critical step in the manufacturing of composite structures, as it directly influences the final quality and performance of the structure [7]. However, this operation represents a major challenge due to the specific characteristics of the Nomex material, considered fragile, and the complex geometry of the honeycomb structure. The unique properties of the NHC structure, such as its light weight and low strength, require special machining methods to avoid potential damage to the structure and ensure optimal results. Additionally, the nature of the material forming the NHC structure makes machining even more difficult, as care must be taken to maintain structural integrity while performing precise cutting operations. Therefore, although machining Nomex honeycomb cores is crucial for manufacturing high-quality composite parts, its complex geometry makes it a major challenge in its practical application. To this end, the ultrasonic vibration machining technique presents itself as an approach capable of reducing cutting forces and optimizing machining quality. This method helps to reduce friction between the tool and the structure, thus generating tangible benefits such as a significant improvement in surface quality and an increased dimensional accuracy of the machined structure. Furthermore, the use of ultrasonic vibration leads to a significant simplification of the complexity inherent in the machining of this structure, thus offering a promising solution to address the challenges associated with this process. The precise control of cutting conditions, such as the frequency, amplitude and direction of ultrasonic vibrations, is of crucial importance in the processing process [8]. This control capability makes it possible to effectively regulate the interaction between the tool and the workpiece, thus leading to a better machinability of the material [9,10,11]. In this context, many researchers have opted for the RUM technology for materials machining. As an example, Zhang et al. [12] conducted a study on elliptical ultrasonic vibration-assisted milling to evaluate the impact of milling amplitude and speed on the surface integrity of Ti-6Al-4V. The obtained results demonstrated a correlation between increasing speed, milling amplitude and increasing surface roughness values. Han et al. [13] examined the use of elliptical ultrasonic vibration milling on titanium alloys. Their study demonstrated that the application of this technique resulted in a significant reduction in the average radial cutting force. Additionally, this method generated significantly finer chips compared to conventional milling conditions, even using the same cutting conditions. In their comparative study, Xie et al. [14] analyzed conventional milling and longitudinal ultrasonic vibration-assisted milling processes to evaluate their impact on the surface integrity of TC18 titanium alloy samples. Their research highlighted the significant influence of rotational speed and vibration amplitude on several key parameters, such as cutting force, cutting temperature, surface topography and surface residual stresses. Until now, the exploration of the ultrasonic vibration machining of the Nomex honeycomb structure has mainly focused on the field of milling, as confirmed by several studies [15,16,17]. A limited number of researchers have also investigated the application of this technique in grinding and drilling [18,19,20]. Numerical research on the ultrasonic vibration milling of Nomex honeycomb composites (NHCs) is currently limited. The integration of numerical methods such as the finite element method is essential for the understanding and optimization of the ultrasonic vibration-assisted milling process. This paper presents a finite element-based 3D numerical model specifically designed for milling the NHC structure, developed using ABAQUS/Explicit software 2017. From this model, two aspects were carefully examined. First of all, this study focused on the selection of the mesh size and the choice of the constitutive law used to carry out the numerical simulations. Then, the second part of this study looked at the influence of the vibration amplitude on various aspects, such as the components of the cutting force, the stress distributions near the cutting zone, the quality of the obtained surface and the size of the chips generated. The obtained results highlight a reduction in the components of the cutting force, an improvement in the quality of the machined surface and a reduction in the size of the chips.

## 2. Materials and Methods

### 2.1. Experimental Parameters

**Figure 1:**
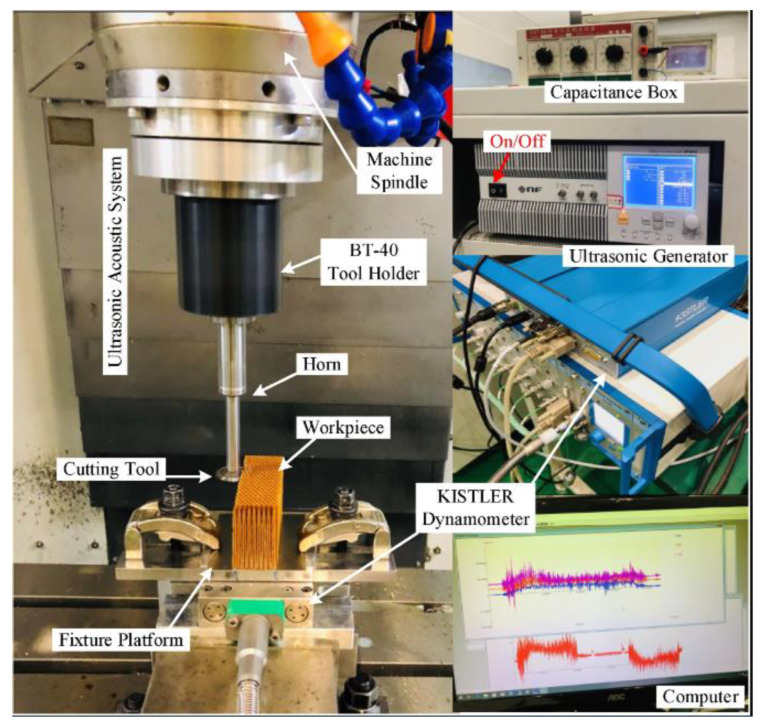
Experimental configuration for rotary ultrasonic machining of Nomex honeycomb composites’ core workpiece [21].

Experimental investigations into the milling of NHC structures utilizing RUM technology were conducted employing the ultrasonic machine tool (THU Ultrasonic 850), developed by Tsinghua University in Beijing, China, as depicted in Figure 1 [21]. The experimental setup includes the following: a BT-40 Tool Holder, an Ultrasonic Acoustic System, an Ultrasonic Generator, a Kistler dynamometer and a Machine Spindle. These elements were brought together in order to collect the essential experimental data during the milling process of the NHC structure.

The instrumentation of the THU Ultrasonic 850 ultrasonic manufacturing device is characterized by several fundamental parameters that influence its operational performance. These parameters, which define the capabilities and limitations of the machine, include the ultrasonic resonance frequency at 20 kHz, the maximum ultrasonic power of 2 kW, the spindle speed reaching up to 10,000 rpm and the 27 μm maximum vibration amplitude of the Resonant Ultrasonic Modulation (RUM) system. These measurements are the primary variables that determine the tool’s ability to perform precise and efficient manufacturing operations in various application contexts.

### 2.2. Material Parameters

**Figure 2:**
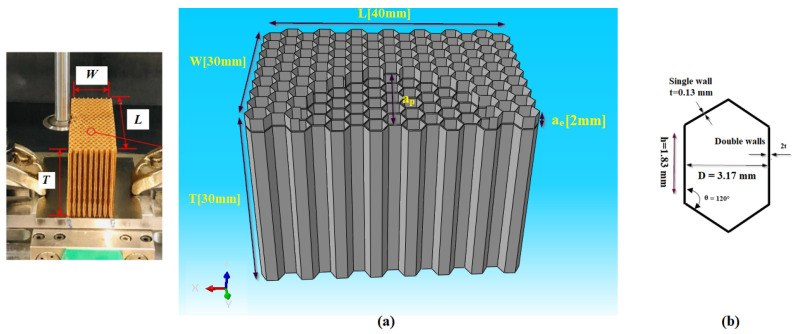
(**a**) The geometric dimensions of the NHC core; (**b**) the geometric dimensions of the hexagonal cell.

The rigorous definition of the properties of the structure to be machined and the cutting tool constitutes a crucial step in the finite element machining simulation process. This approach is of fundamental importance to ensure the precision and reliability of the obtained results. Therefore, it is imperative to precisely define the mechanical and geometric properties of the structure and the cutting tool during finite element modeling. As part of this study, the workpiece, namely the NHC structure, was designed according to the established experimental procedure [21]. The geometric dimensions of the NHC structure as well as the cell are listed in Figure 2.

**Figure 3:**
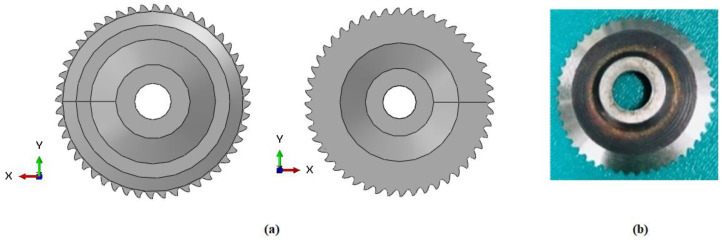
The presentation of the UCSB cutting tool used to cut the NHC core: (**a**) UCSB designed by simulation; (**b**) UCSB used in the experiment.

**Figure 4:**
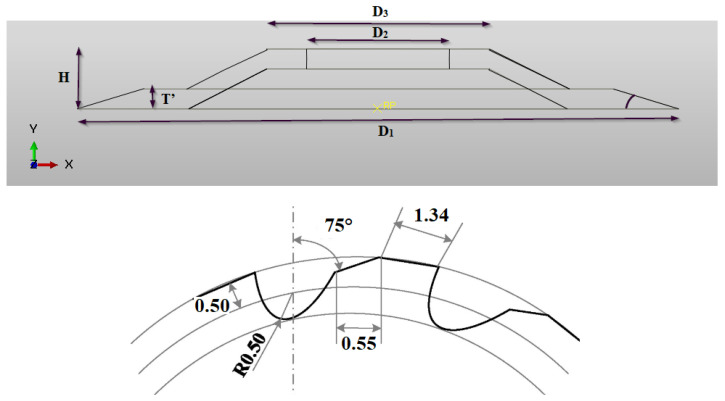
The dimensions of the UCSB cutting tool.

According to the experimental procedure, the NHC core was machined using a specific cutting tool designated UCSB, which is a high-speed steel (HSS-W18Cr4V) ultrasonic circular milling cutter (see Figure 3) [21]. To conduct this, the cutting tool was designed taking into consideration the geometric dimensions previously used during the experimental phase (Figure 4).

### 2.3. Constitutive Model

The machining of composite materials results in a process where the material undergoes significant deformation under the impact of a cutting tool, which leads to the formation of chips. This process is characterized by high levels of strain, strain rate and temperature. Thus, the precise establishment of the law of behavior of the workpiece and the cutting tool constitutes an essential prerequisite for carrying out numerical simulations using the finite element method. Generally speaking, composite materials are recognized for their orthotropic behavior. However, the approach taken in this study differs, where the material forming the Nomex honeycomb structure is defined by brittle isotropic elastoplastic behavior, as stipulated by previous work by Foo and Ray [22,23]. The elastoplastic behavior is based on a decomposition of the total deformation into two distinct components: an elastic part, which is reversible, and a plastic part, which is irreversible according to the following equation [24].

(1) $$\varepsilon =\varepsilon^{el}+\varepsilon^{p}$$

where *ε* is the total stress tensor, and *ε^el^* and *ε_p_* are the elastic and plastic strain tensors, respectively.

Generally, elastic behavior is characterized using Hooke’s law which is presented in the form of a linear elasticity model. The elastic part obeys Hooke’s law, expressed by the following equation [24]:

(2) $$\left\{\varepsilon \right\}=\left[C\right]\left\{\sigma \right\}$$

It is the tensor of the reasoner of order 4.

The law therefore becomes the following:

(3) $$\left(\begin{array}{c} \varepsilon_{11}\\ \varepsilon_{22}\\ \varepsilon_{33}\\ \varepsilon_{23}\\ \varepsilon_{31}\\ \varepsilon_{12} \end{array}\right)=\left(\begin{array}{cccccc} \frac{1}{E} & -\frac{\vartheta }{E} & -\frac{\vartheta }{E} & 0 & 0 & 0\\ -\frac{\vartheta }{E} & -\frac{1}{E} & -\frac{\vartheta }{E} & 0 & 0 & 0\\ -\frac{\vartheta }{E} & -\frac{\vartheta }{E} & \frac{1}{E} & 0 & 0 & 0\\ 0 & 0 & 0 & \frac{1}{G} & 0 & 0\\ 0 & 0 & 0 & 0 & \frac{1}{G} & 0\\ 0 & 0 & 0 & 0 & 0 & \frac{1}{G} \end{array}\right)\left(\begin{array}{c} \sigma_{11}\\ \sigma_{22}\\ \sigma_{33}\\ \sigma_{23}\\ \sigma_{31}\\ \sigma_{12} \end{array}\right)$$

For a complete characterization of the elastic behavior of the material, it is essential to calculate the Lame coefficients λ and µ. The relationships between the Lame coefficients λ, µ, the Young Modulus E and the Poisson ratio ν are expressed by the following equations [24]:

(4) $$\lambda =\frac{\vartheta \cdot E}{\left(1+\vartheta \right)\left(1-2\vartheta \right)}$$

(5) $$µ=\frac{E}{2\;\left(1+\vartheta \right)}$$

**Figure 5:**
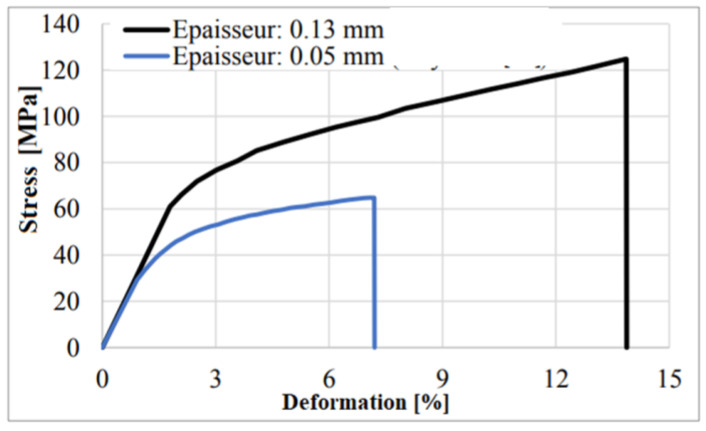
Stress–deformation curves for Nomex paper [22,23].

Several authors have associated an isotropic elastoplastic behavior law with Nomex paper [25,26]. Figure 5 illustrates the mechanical behavior of Nomex paper as determined by Foo et al. [22] and Roy et al. [23]. The curves present elastic thresholds as well as varied plastic behaviors depending on the thickness of the walls.

As a result, the elastoplastic behavior of the walls of the honeycombs will be conditioned by their thickness, whether it is a single or double wall. The main mechanical properties assigned to Nomex paper are listed in Table 1. The yield stresses for each wall thickness are defined as σ_0_ = 29 MPa and σ_1_ = 61 MPa for single and double walls, respectively. Consequently, the maximum allowable stresses are σ_max0_ = 64.2 MPa and σ_max1_ = 124.8 MPa for single and double walls, respectively.

### 2.4. Chip Separation Criterion

The Nomex honeycomb composite (NHC) structure machining procedure involves cutting the material under the influence of a cutting tool. In this context, the determination of the failure criterion is of essential importance in the machining simulation process. The separation criteria are generally correlated to the mechanical and thermal properties intrinsic to the material. In the context of this study, the shear failure criterion is specifically adopted, and its control is entrusted to the finite element software Abaqus. The damage analysis procedure begins with the synthesis of the initial mechanical properties, as shown in Table 1. Then, the movement of the cutting tool generates a force applied to the workpiece, with the calculation of dynamic normal and shear stresses carried out at the different interfaces. Thus, to precede the initiation and propagation of damage over several sequences, the appropriate breakage criteria are applied. When the fracture coefficient, ω, reaches a critical threshold of 1, signaling material degradation and chip formation, this value can be determined using the following equation [27]:

(6) $$\omega =\sum \frac{∆\varepsilon }{\varepsilon^{f}}$$

where *ω* is the shaping limit value or destruction coefficient, ∆*ε* is the equivalent plastic strain increment and *ε^f^* is the total strain when the material is damaged.

### 2.5. Simulation Model

**Figure 6:**
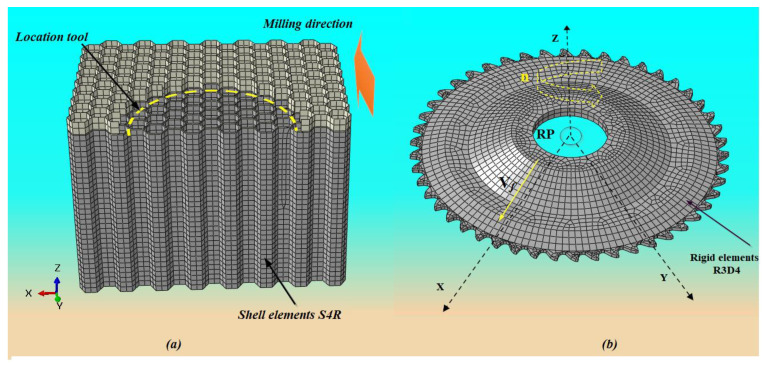
(**a**) The mesh used in the FE model; (**b**) the cutting conditions defined at reference point RP: n is the spindle speed, and V*_f_* is the feed rate.

In this paper, a numerical study of the milling process of the NHC structure is proposed using RUM technology, where the structure to be machined is made up of thin walls of low thickness. From this perspective, the mesh of the walls of the NHC structure is chosen using classic shell elements (S4R) with four nodes, assigning to each node six degrees of freedom (three translations and three rotations) with a reduced integration method (Figure 6a). In the context of the numerical simulation, the cutting tool is considered to be rigid, which implies that it remains free of any deformation throughout the machining operation. In order to obtain a precise representation in the numerical model, it is discretized using rigid quadrangular elements with four nodes (R3D4) (Figure 6b).

**Figure 7:**
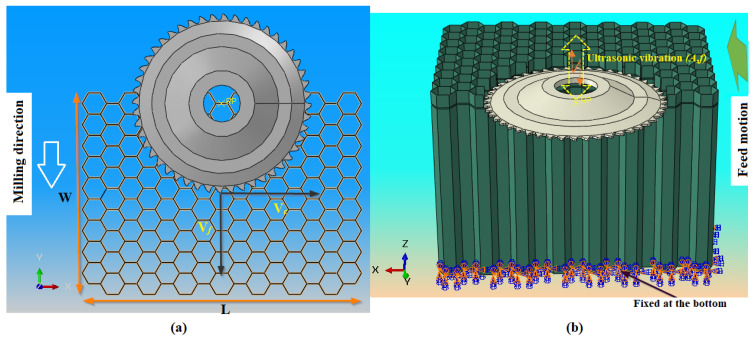
(**a**) NHC structure milling process plan; (**b**) boundary conditions used in numerical simulation.

Before launching the execution of the numerical code, it is essential to carry out a preliminary phase in order to analyze the non-linear parameters which could have an impact on the convergence of the numerical results. Various factors must be taken into consideration during numerical simulation, including mesh size. It is important to note that using a small mesh size can result in a significant increase in calculation time (CPU), especially in the case of three-dimensional configurations. Thus, it becomes essential to find an optimal balance between the size of the mesh and the precision of the results while respecting a reasonable calculation time. During the milling simulation, a general contact was used to represent the interaction between the cutting tool and the walls of the Nomex honeycomb composite (NHC) structure. Given the thin walls of the NHC structure, the contact between the cutting tool and the NHC structure is treated as a point contact governed by Coulomb’s law. Therefore, the contact coefficient adopted for the numerical simulations is 0.1. In order to ensure full contact between the cutting tool and the NHC structure from the start of the simulation, an initial engagement configuration was created, taking into account the specific geometric characteristics of the honeycomb structure and the cutting tool (Figure 6a). Regarding the boundary conditions, they were adopted according to the established experimental procedure [21]. In this context, the bottom surface of the NHC structure remains fixed, indicating that it is immobile and cannot be subjected to any translation or rotation. In our numerical approach, full embedding is applied to the bottom surface of the NHC core. This results in the elimination of any translation (U_x_ = U_y_ = U_z_ = 0) along the X, Y and Z axes, as well as any rotation (U_Rx_ = U_Ry_ = U_Rz_ = 0) around the X, Y and Z axes, as shown in Figure 7b. The rotary ultrasonic machining (RUM) process is a mechanical manufacturing method for removing material, based on the coordination of three movements of the cutting tool. These movements include the feed movement along the axis OY, characterized by the feed speed V*_f_*, the rotation of the cutting tool around the axis OZ characterized by a rotation speed denoted *n* and the movement vibration along the OZ axis, generating a sinusoidal ultrasonic wave (Figure 7a). To accurately monitor the machining process during numerical simulation, a reference point designated as RP was assigned on the rotation axis of the cutting tool, as shown in Figure 6b. This RP is essential for establishing the cutting conditions and evaluating the cutting forces during the milling process. The movement of the cutting tool can be described in terms of global coordinates *xyz* by the following equations [8]:

(7) $$V_{x}=V_{c}\;cos\;\left(\;\frac{2\pi n}{60}t\right)+V_{f}$$

(8) $$V_{y}=V_{c}\;sin\;\left(\;\frac{2\pi n}{60}t\right)$$

(9) $$V_{z}=A\;sin\;(2\pi ft)$$

*A* represents the ultrasonic vibration amplitude, *V_c_* is the cutting speed, *V_f_* is the feed rate, n is the spindle speed, *t* is the time and *f* is the vibration frequency, which is 22.050 KHz in the present paper.

### 2.6. The Formulas for the Components of the Cutting Force

During the experimental phase, the cutting force components, namely *F_y_* (feed component along *Y*) and *F_x_* (cutting component along *X*), are measured using the KISTLER-9256C2 dynamometer. A benefit of this technique is that it is possible to calculate the average value in both directions using the following formulas [8,21]:

(10) $$F_{x}=\frac{1}{t_{2}-t_{1}}\;\int_{t_{1}}^{t_{2}}\left|F_{CX}\right|\;dt$$

(11) $$F_{y}=\frac{1}{t_{2}-t_{1}}\;\int_{t_{1}}^{t_{2}}\left|F_{CY}\right|\;dt$$

*F_x_* and *F_y_* express the averages of the cutting force components along the *X* and *Y* axes, while *t*_1_ and *t*_2_ denote the start and end of the cutting process, respectively.

## 3. Results and Discussion

The deformation of the NHC core during milling can impact the cutting force and the quality of the machined surface. This deformation is mainly caused by the low rigidity of the Nomex paper constituting the NHC core. However, the NHC structure exhibits high stiffness in the out-of-plane direction (T direction) along the z-axis. Thus, the compression component F_z_ has no major impact on the milling process. In this context, research focuses on the feed component F_y_ and the cutting component F_x_. At the same time, this article presents the influence of the amplitude of vibrations on the quality of the machined surface, the size of the chips generated and the distribution of stresses in the cutting zone.

### 3.1. Mesh Study

**Figure 8:**
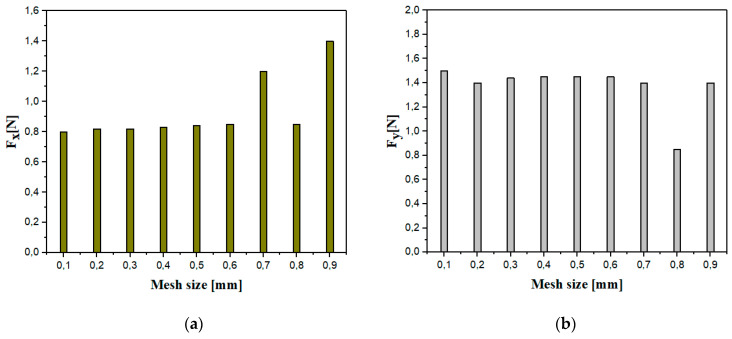
The components of the cutting force for different mesh sizes: (**a**) the cutting component *F_x_*; (**b**) the advance component *F_y_*.

The selection of the mesh size constitutes a fundamental element in the field of numerical simulation. This is explained by its direct impact on the precision of the results and on CPU calculation time. Regarding the fine mesh, using the finite element formulation with full integration results in a longer calculation time but ensures more reliable results. On the other hand, with a coarse mesh, although the calculation time is reduced, the results are not precise. Thus, in each analysis, it is essential to seek an optimal balance between the size of the mesh, the CPU calculation time and the precision of the obtained results. To appropriately choose the mesh size for numerical simulations of the milling of the NHC structure assisted by ultrasonic vibrations (RUM), numerical studies were carried out in order to analyze the impact of this size on the components of the cutting force *F_x_* and *F_y_*. To accomplish this objective, nine simulations were carried out, covering a range of meshes extending from 0.1 to 0.9 mm respecting the same cutting conditions including a spindle speed of 3000 rpm, a feed speed of 2000 mm/min, a cutting depth of 2 mm and a vibration amplitude of 25 µm. The results of these analyses are presented in Figure 8.

The main observations highlighted an instability of the components of the cutting force F_x_ and F_y_, when the mesh size is between 0.7 and 0.9 mm. More specifically, a decrease in the F_x_ and F_y_ components was noted between mesh sizes of 0.7 mm and 0.8 mm, followed by an increase between 0.8 mm and 0.9 mm. This discrepancy can be attributed to the high sensitivity to the elastic restoring force of the walls. As the tool progresses through the material, when an element reaches its breaking point, it becomes detached, causing a loss of contact between the material and the cutting tool. This loss of contact reduces the cutting forces. In reality, the resistance generated by the elastic return of the intact walls creates a force opposing the rotation of the tool, which results in an effective increase in the cutting force components. On the other hand, the results indicate a stabilization of the *F_x_* and *F_y_* components when the mesh size varies between 0.1 and 0.6 mm. However, small elements have a reduced ability to resist deformation. As a result, mesh distortion issues tend to manifest more quickly, causing calculations to break. In principle, the optimal size mesh established at 0.2 mm ensures both an adequate precision of the results and reasonable calculation efficiency. However, a re-evaluation is now necessary, leading to adjusting the mesh size to 0.4 mm. This modification is undertaken in order to achieve an optimal compromise between the precision of the results and the reasonable calculation time while avoiding the distortion of the elements.

### 3.2. The Identification of the Behavioral Law Used in Numerical Modeling

**Figure 9:**
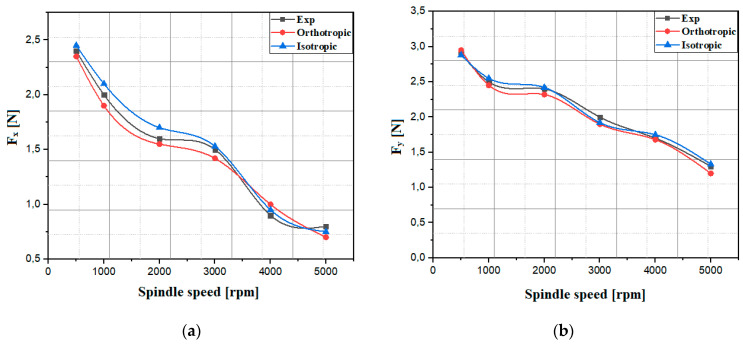
The components of the cutting force for different spindle speeds: (**a**) the cutting component *F_x_*; (**b**) the advance component *F_y_*.

For an in-depth analysis of the simulation of milling assisted by ultrasonic vibrations of the Nomex honeycomb composite (NHC) structure, it is essential to take into account the law of behavior of the material composing the structure. Due to the complexity of the composition of the Nomex paper constituting the walls of the NHC structure, two mechanical behaviors were taken into consideration, on the one hand, an elastoplastic–isotropic behavior, representing the deformation properties of the material without taking into account its orientation, on the other hand, an orthotropic behavior associated with the Tsai–Wu rupture criterion, thus making it possible to reflect its specific characteristics according to different orientations. In this context, numerical simulations were carried out to study the impact of the cutting tool spindle speed on the *F_x_* and *F_y_* components. The simulations are based on six distinct rotation speeds, including 500 rpm, 1000 rpm, 2000 rpm, 3000 rpm, 4000 rpm and 5000 rpm. This was achieved by keeping the other cutting conditions constant, namely a constant feed rate of 500 mm/min and a vibration amplitude of 25 µm. The obtained numerical results by the two proposed approaches were compared to those obtained by the experiment, as indicated in Figure 9 [21].

The results from the two proposed approaches generally follow the experimental trend observed for the cutting force components, which generally decrease with increasing spindle speed. It is clearly observed that the components of the cutting force *F_x_* and *F_y_* are significant at low rotation speeds, this phenomenon being attributable to the accumulation of chips in front of the cutting tool. The mechanical characteristics of Nomex paper help to maintain this accumulation due to the elastic deformation of the walls, before they reach the breaking point. However, when the spindle speed reaches excessive levels, the contact between the cutting tool and the workpiece material per unit time becomes excessively pronounced. This strong contact generates undesirable heating phenomena, thus inducing thermal instability within the cutting system [28]. Furthermore, the low contact between the tool tooth of the UCSB tool and the honeycomb wall is a crucial aspect, as it reduces local stresses and contributes to better heat dissipation. The honeycomb geometry of the NHC structure promotes more efficient air circulation through the structure, which helps to dissipate heat generated during the machining process. Due to the complexity associated with the orthotropic elastoplastic approach, which requires the proper integration of each component of the Nomex paper, it is more judicious to adopt the isotropic elastoplastic approach, due to its easier implementation in terms of a more reasonable calculation time.

### 3.3. Effect of Cutting Width on Components of Cutting Force

**Figure 10:**
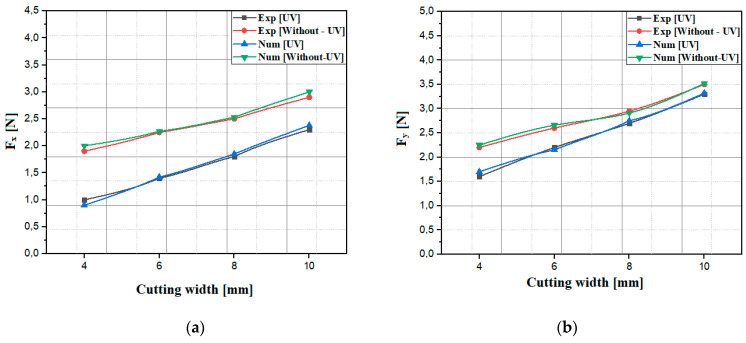
The components of the cutting force for different cutting widths: (**a**) the cutting component *F_x_*; (**b**) the advance component *F_y_*.

To validate the developed numerical model, an analysis of the impact of different cutting widths on the F_x_ and F_y_ components during the milling of the NHC structure was carried out, using RUM technology, whether with the application of ultrasonic vibration (UV) or without it. To conduct this, four cutting width values were provided during the simulation, namely 4 µm, 6 µm, 8 µm and 10 µm, while the other cutting conditions remained constant throughout the simulation such as, notably, the feed speed at 3000 mm/min and the rotation speed at 5000 rpm. The obtained results are compared with the experimental results and are presented in Figure 10 [21].

The numerical simulation results agree with the experimental trend observed for the *F_x_* and *F_y_* components, highlighting a notable increase in the cutting width when milling the NHC structure, independent of the application of ultrasonic vibrations. This increase in the cutting width results in an increase in the contact surface between the cutting tool and the part, thus leading to an increase in the flow rate of the material removed per unit of time. Furthermore, this increase can cause an increase in friction resistance during the machining process, thus leading to an intensification of cutting forces. During the cutting operation of the NHC structure using RUM technology, it is evident that the application of ultrasonic vibrations induces a notable reduction in the *F_x_* and *F_y_* components, reaching a significant reduction of 50%. In this context, the intense rotation and vibration of the cutting tool promote the formation of cracks in the walls of the NHC structure, thus facilitating the penetration of the tool without encountering resistance from the material constituting the NHC structure. Although a larger cutting width can lead to better cutting efficiency, it is also associated with an increased cutting force, which can lead to other problems such as the premature wear of the cutting tool and degradation of the quality of the machined surface. Therefore, it is essential to set an optimal value of the cutting width to strike a balance between these two aspects when milling the NHC structure. This optimum value maximizes the volume of the material removed while maintaining the cutting force components at an acceptable level, thereby ensuring satisfactory cutting efficiency and optimal processing performance. In general, the results from the numerical simulation show a significant correlation with the experimental data, which underlines the robustness of the developed numerical model.

### 3.4. The Influence of the Vibration Amplitude on the Quality of the Machined Surface

**Figure 11:**
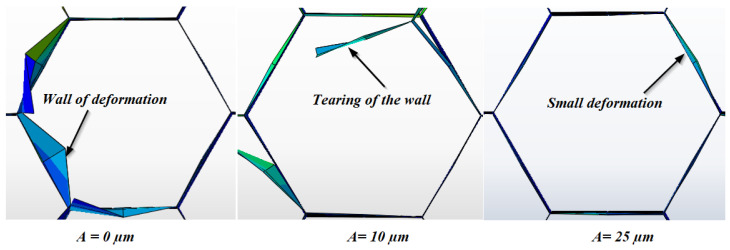
Analysis of NHC surface morphology after machining.

The quality of the machined surface is of paramount importance in the manufacturing of sandwich materials. Thus, the precise optimization of this quality is essential to guarantee the reliability and optimal performance of these structures in a varied range of industrial applications. In general, machining defects of the NHC structure are manifested by various irregularities such as burrs and tears of the uncut fiber. These imperfections can be observed throughout the machining process and are likely to influence the final quality of the structure, thus requiring an in-depth analysis to optimize manufacturing parameters and minimize defects. To evaluate the impact of the vibration amplitude on the machined surface quality, numerical simulations were carried out by varying the vibration amplitude, including 0 µm, 10 µm and 25 µm. The other cutting conditions remained constant throughout the milling simulation, notably the feed speed at 3000 mm/min and the rotation speed at 2000 rpm. Machining defects resulting from the numerical model were identified based on visual examination with the naked eye and are shown in Figure 11.

The main finding from this study lies in the improvement in the quality of the generated surface as the amplitude of ultrasonic vibrations increases. This observation suggests a direct link between vibration amplitude and machined surface characteristics, highlighting the beneficial potential of ultrasonic vibrations in the machining process. Furthermore, the numerical results indicate that the main machining defects observed on the NHC core are the deformations and tears of the walls. However, burrs characterized by excess material on the cell walls were not detected by the numerical model, given that these were represented by S4R shell elements without thickness. In this context, at relatively low spindle speeds, the cutting tool cannot exert sufficient pressure on the walls of the NHC structure, thus increasing the risk of the elastic deformation of these walls until their rupture. The integration of ultrasonic vibrations highlights the significant benefits of ultrasonic vibrations in the cutting process. These vibrations act by reducing contact between the tool cutting edge and the thin walls of the NHC structure, resulting in reduced friction and improved heat dissipation during cutting. These factors facilitate the penetration of the cutting tool into the walls of the NHC structure, thus contributing to the reduction in machining defects and the improvement in the quality of the machined surface. Indeed, this highlights the ability of ultrasonic vibrations to improve the quality of the machined surface, which could have significant implications for the optimization of manufacturing processes.

### 3.5. The Repartition of Stresses in the Cutting Zone

**Figure 12:**
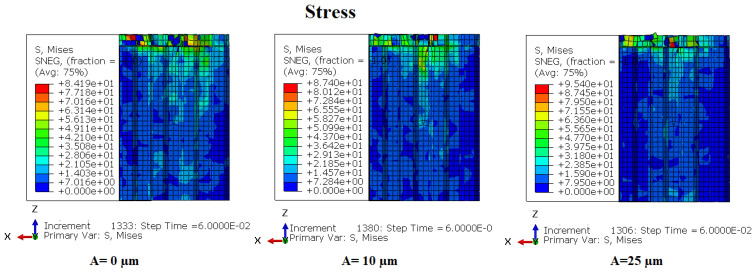
Comparison of stresses and Mises displacements under different amplitudes.

**Figure 13:**
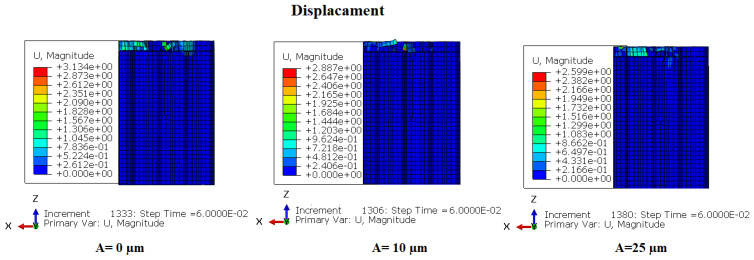
Comparison of Mises displacements under different amplitudes.

In this section, numerical simulations were conducted to analyze the effect of the vibration amplitude of the cutting tool on the stress distribution during the milling of the NHC structure. The simulations consisted of varying the vibration amplitudes of the cutting tool, namely 0 µm, 10 µm and 25 µm, while the other cutting conditions remained constant, namely the feed speed at 3000 mm/ min, the rotation speed at 3000 rpm and the cutting depth at 2 mm. The obtained results are presented in Figure 12 and Figure 13.

The analysis of the stress distribution on the honeycomb walls of the NHC structure provides significant insight into the cutting condition near the machining zone, providing an in-depth understanding of the impact of ultrasonic vibrations during the process cutting. The figures present a simultaneous comparison of the stresses and displacements of honeycomb cell walls under different amplitudes and at the same time. In order to improve the visualization of the stresses and deformations of the cell walls, the cutting tool was not included in the representations. Under the effect of high amplitudes, failure has already occurred due to the large stresses required to cause failure in accordance with the established failure criterion. Elements with stresses below the failure limit are visible, while those with higher stresses have been shredded and are not noticeable. For an amplitude of 0 μm (without ultrasonic vibration), the maximum stress on the part reaches 84.19 MPa, a value lower than that observed with amplitudes of 10 μm and 25 μm. However, the cell wall displacement is significantly greater than that observed during ultrasonic cutting. Thus, during the ultrasonic cutting process of Nomex honeycomb, an increase in the amplitude of the ultrasound leads to a faster reaching of the ultimate strength of the cut cell wall, thus allowing for cutting without major deformations or significant damage. In this regard, studies by Sun et al. [29] on the ultrasonic cutting of aluminum honeycombs, as well as those of Arnold et al. [30] on ultrasonic food cutting, reached similar conclusions. This research demonstrated that the use of ultrasonic vibrations in the cutting process leads to comparable results, highlighting the effectiveness and benefits of this approach in varied contexts.

### 3.6. The Influence of the Vibration Amplitude on the Size of the Chips Generated

**Figure 14:**
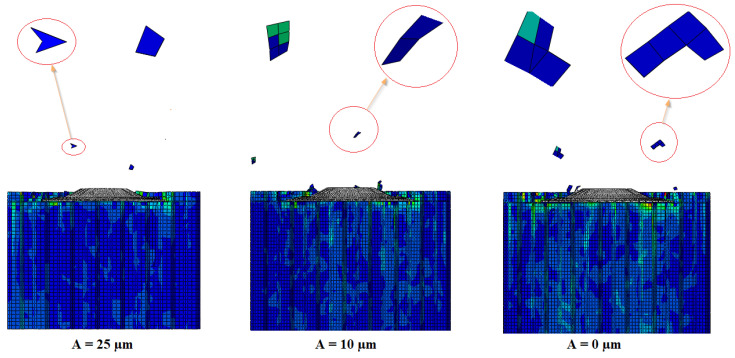
Chip size for different vibration amplitudes.

This paragraph proposes numerical simulations analyzing the effect of the amplitude of the vibrations on the size of the chips generated during the cutting of the NHC structure assisted by ultrasonic vibrations. To conduct this, three amplitudes were examined, namely 0 µm, 10 µm and 25 µm. It should be noted that the other cutting conditions remained constant throughout the numerical simulation, including a feed speed of 3000 mm/min, a spindle speed of 5000 rpm and a cutting depth of 2 mm. The obtained results are presented in Figure 14.

In fact, the milling of the NHC structures takes place in two distinct stages. First, the milling cutter cuts the thin walls of the NHC structure. Then, the cut parts progress towards the upper part of the tool, where they undergo a process of shredding and regrowth due to the high rotation speed, thus giving rise to the chips. Based on the obtained results, it is evident that the resulting chip size during the ultrasonic vibration-assisted milling of the Nomex honeycomb structure is extremely related to the amplitude of ultrasonic vibrations. Increasing the amplitude of ultrasonic vibrations results in a significant reduction in chip size. This observation is explained by the fact that high vibration amplitudes combined with a high rotational speed reduce the contact between the edge of the cutting tool and the walls of the NHC structure, which facilitates the penetration of the tool in the thin wall. This reduction in contact then intensifies the propagation of cracks within the structure. As a result, the walls of the structure are cut more efficiently, thus favoring the formation of small chips. Ultimately, the reduction in chip size helps reduce cutting forces, thus improving the quality and efficiency of the milling process, thus avoiding the premature wear of the cutting tool.

## 4. Conclusions

In the present paper, a numerical FE model of the ultrasonic vibration-assisted milling of the Nomex honeycomb composite (NHC) structure was developed and validated with a corresponding experiment. Firstly, an appropriate mesh size was selected in order to optimize the compromise between the CPU calculation time and the reliability of the results. The mesh size chosen for this study is 0.4 mm. After defining the appropriate mesh and identifying the constitutive laws used for the numerical simulations, a finite element analysis was undertaken to examine the effect of the cutting width on the cutting force components *F_x_* and *F_y_*, as well as the influence of the amplitude of vibrations on the quality of the machined surface, the size of the chips generated and the distribution of stresses in the cutting zone. From this work, the following conclusions can be drawn:

- The effect of the cutting width on the *F_x_* and *F_y_* components demonstrates an almost proportional increase, confirmed by simulations and experiments, resulting from the larger contact surface between the tool and the workpiece, as well as an increased volume of material removed per unit of time.
- Increasing the amplitude of ultrasonic vibrations during milling results in a noticeable reduction in chip size due to a decrease in contact between the cutting tool and the walls of the NHC structure.
- Increasing the amplitude of ultrasonic vibrations is essential to improve the quality of the machined surface, revealing their beneficial potential in the machining process; their integration reduces machining defects by facilitating cutting tool penetration, which could have significant implications for the optimization of manufacturing processes.
- When the cutting tool is subjected to ultrasonic vibration, increased stress is induced in the cutting area of the honeycomb cell wall. This results in rapid material breakdown and reduced cell wall deformation, allowing for a smoother cutting of the Nomex honeycomb structure.
- From an industrial point of view, optimizing machining operations requires prolonged and costly experiments to test various configurations. The proposed 3D modeling can thus be very beneficial in terms of speed and efficiency while being economical.
- It should be noted that numerical modeling may have limitations in terms of the accuracy and validity of the results, requiring experimental validation to ensure the reliability of predictions.

## Figures and Tables

**Table 1:** The mechanical properties of Nomex paper [22,23].

Density [g/cm^3^]	Young Modulus [MPa]	Poisson’s Ratio
1.4	3400	0.3

## Data Availability

Data are contained within the article.
